# Factors Influencing Occupation-Based Practice in Physical Dysfunction: Perspectives of Thai Occupational Therapists

**DOI:** 10.1155/oti/9955358

**Published:** 2025-12-03

**Authors:** Patcharawalai Tupsai, Peeraya Munkhetvit, Anuchart Kaunnil

**Affiliations:** Department of Occupational Therapy, Faculty of Associated Medical Sciences, Chiang Mai University, Chiang Mai, Chiang Mai Province, Thailand

**Keywords:** client-centered practice, mixed methods, occupation, occupational therapy

## Abstract

**Introduction:**

Occupation-based practice (OBP) is a core concept in occupational therapy, integrating meaningful and purposeful occupations into assessment, intervention, and outcomes. There were 561 Thai occupational therapists who specialize in treating clients with physical dysfunction who participated in this study. This study was aimed at examining the perspectives and experiences of occupational therapists using OBP for clients with physical dysfunction.

**Method:**

In the survey phase, there was an online survey conducted using a Google survey found on a social media platform that was listed as the official website, and the results were analyzed using descriptive statistics. In the interview phase, nine participants were interviewed, and their responses were analyzed through thematic analysis.

**Results:**

Sixty-two participants responded. Quantitative and qualitative results were integrated. Forty-seven percent strongly agreed, and 39% agreed that allowing clients to choose relevant occupations was essential. Fifty percent strongly agreed and 40% agreed that OT services should be based on real-life contexts. OBP was also used to encourage clients to pursue more occupations (47% strongly agreed, 48% agreed). The interviews support the notion that attitudes toward occupation drive OBP. When addressing the role of facilitators, 37% strongly agreed and 47% agreed that clients were key in enabling OBP. Therapists found OBP effective for treatment (36% strongly agreed, 45% agreed). Barriers included insufficient time for OBP (21% strongly disagreed, 39% disagreed) and that there are inadequate clinical settings (23% strongly disagreed, 34% disagreed). These findings concurred with interview data which highlighted limited time and inadequate space as key factors influencing OBP.

**Conclusion:**

The core features of OBP are client-centered goals, implementation, motivation, and engagement, highlighting the importance of occupation in practice. Influencing factors include clients, therapists, policies, service systems, and clinical settings. When implementing OBP, therapists should consider these factors and their relationships.

## 1. Introduction

Occupational therapy has made significant contributions to healthcare in recent decades due to its unique philosophical concept of occupation [1]. Expert occupational therapists emphasized that occupational therapy has reestablished occupation as its core organizing concept, reflecting a contemporary paradigm in practice [2–4]. This approach encompasses not only assessment but also intervention and outcome-focused strategies to empower clients in meeting their needs [5]. Hence, occupation is the primary means and end of occupational therapy interventions [6, 7]. First, occupations are fundamentally linked to health and wellness. Second, clients may face occupational dysfunctions resulting from various challenges and problems. Third, occupation-based practice (OBP) serves as a treatment approach [2]. According to Fisher [8], OBP is the assessment, intervention, and outcome of the occupational therapy process.

Despite its importance, the focus on occupation and its role in therapy has not always been a central part of practice, both throughout history and more recently [9]. OTs still face challenges in placing occupation as the central focus of their clinical services, using it as both a goal-directed action and a primary therapeutic agent [8]. This is consistent with Hess-April et al. [10], who found tension between medical models and a holistic approach among OTs in hospitals and rehabilitation centers. Therefore, OTs are concerned not only with the occupations of their clients but also with various factors that facilitate or hinder their occupations and impact their participation and engagement [11]. Using OBP to enhance the uniqueness of OTs and improve the clinical outcomes and occupational performance of clients has become a vital part of occupational therapy practice in the contemporary paradigm [12].

While OBP in contemporary Western contexts, as described by Kielhofner [2], often emphasizes individual autonomy, volition, and self-directed goals, these principles do not always translate seamlessly into Eastern cultural settings such as Thailand. In many Asian societies, including Thailand and Malaysia, collectivist values and hierarchical social structures shape healthcare delivery and the dynamics between clients and therapists. For example, Daud et al. [13] defined occupation-based intervention in Malaysia as an approach that enhances occupational performance by aligning therapeutic activities not only with clients' personal goals but also with culturally meaningful occupations.

Thai culture, as part of the Asian and non-Western tradition, is characterized by collectivism, an interdependent self-construal, and strong family involvement in decision-making [14]. Embedding occupations that reflect Thai cultural values is therefore an effective way to promote OBP among occupational therapists. For instance, stroke rehabilitation in Thailand extends beyond focusing solely on the individual client; implementing OBP necessitates integrating family perspectives and concerns for the sociocultural norms that influence decision-making and therapeutic participation.

Additionally, Thailand's public health services follow a hierarchical structure that extends from the central to the subdistrict level. At the apex are university-affiliated medical colleges or faculties of medicine, followed by regional hospitals, provincial hospitals, district hospitals, and finally subdistrict hospitals [15]. This hierarchy, combined with associated healthcare policies, can restrict therapists' professional autonomy, particularly in making OBP-related decisions. In regional and provincial hospitals, shorter patient stays often limit opportunities to implement OBP for clients with physical dysfunction, with interventions frequently centered on medical management and impairment reduction. By contrast, district and subdistrict hospitals, as well as community rehabilitation centers, are situated closer to clients' homes and operate under policies that promote participation in daily life. These settings provide more scope for OBP, enabling therapists to design interventions that leverage local materials, community resources, and family involvement.

Nonetheless, public understanding of OBP in Thailand remains limited [16], and the term itself is often unfamiliar to clients and other healthcare providers. These cultural and systemic factors underscore the importance of adapting OBP for the Thai context, ensuring that practice aligns with local values, facilitates shared decision-making with families, and navigates structural hierarchies that may otherwise restrict the participation of clients with physical dysfunction in meaningful occupations.

Implementing OBP can enhance occupational performance, motor and psychological functions, and social participation among adults with physical dysfunction, including those with spinal cord injuries and stroke [17–19]. However, despite recognizing OBP's importance, occupational therapists often encounter barriers such as time constraints, limited resources, insurance demands, inadequate interdisciplinary support, and insufficient training, particularly in hand therapy and hospital settings [20–22]. Consequently, although OBP is closely linked to occupational therapy, its application in clinical practice remains limited [13, 17–19, 21–23].

In Thailand, rehabilitation wards primarily treat patients with neurological conditions, including stroke, spinal cord injury, and traumatic brain injury [22, 24]. Additionally, musculoskeletal and connective tissue disorders have become increasingly common reasons for outpatient visits in public hospitals [25]. A study in Thailand indicated that most occupational therapists view OBP as a core principle of their profession. However, an imbalance between a shortage of OTs, high caseloads, cultural and family beliefs, the mindset of teams, and the need for appropriate and contextual settings impedes OBP implementation [16]. Moreover, studies have only covered general OT practice areas without focusing on the field of physical dysfunction in treatment services.

As practitioners, OTs play an essential role in developing the skills and abilities of clients in hospitals and clinics who have physical dysfunctions. However, there are limited studies on the role that Thai OTs play in providing OBP to adults with physical dysfunctions. Therefore, our objective was to explore the opinions and experiences of Thai OTs regarding OBP in clients with physical dysfunction. This study involved identifying the facilitators and barriers affecting OBP. This included the impacts of failure to incorporate OBP for this population related to assessment, intervention, and client outcomes.

## 2. Methods

### 2.1. Study Design

An explanatory sequential mixed method design was used [26], consisting of two phases. First, quantitative data was collected through a cross-sectional survey and was analyzed. Based on these quantitative findings, qualitative data was also collected and analyzed, and this qualitative data provided further explanation of the quantitative results. Therefore, this quantitative study was aimed at exploring the perspective of OTs using OBP in such clients. A qualitative descriptive study was used to investigate the experiences of Thai OTs using OBP for physically dysfunctional clients, employing semistructured interviews for data collection. The research design, methodology, methods, and analysis were carefully considered, and rigor was ensured in this study [27]. This study received ethical approval from the Ethics Committee of Faculty of Associated Medical Sciences, Chiang Mai University (Reference Number AMSEC-66EX-005).

### 2.2. Participants and Recruitment

#### 2.2.1. Survey Participants

There are 1674 licensed occupational therapists in Thailand, 561 of whom specialize in treating clients with physical dysfunctions, according to the Occupational Therapist Association of Thailand [28]. The participants were selected from survey data using convenience sampling. The participants were recruited based on the following criteria: (1) possessed a national occupational therapy license, (2) worked with clients who had physical dysfunction, and (3) had more than 1 year of experience using OBP for clients with physical dysfunction. Advertisements and contact forms were sent to the OTs via mail and online via Google Forms.

#### 2.2.2. Sample Size Calculation

The sample size for this study was determined using Yamane's [29] formula for finite population sampling, which is appropriate for quantitative studies with a clearly defined population size. Based on the total population of 561 OTs working with clients with physical dysfunction and adopting a confidence interval of 90% with a significance level of 0.1, the calculated sample size was 85 respondents.

#### 2.2.3. Survey Procedure

The questionnaire survey was developed based on observed and reported therapeutic interventions within occupational therapy services in Thailand [16], aiming to comprehensively examine the construct of OBP. The survey consisted of four major sections. (1) General information: including participants' age, years of experience, level of education, and workplace setting (e.g., community hospitals, general hospitals, or rehabilitation centers). (2) Experience using OBP: participants were asked to indicate how often they used OBP in practice, the populations they applied it to, and their training background related to OBP. (3) Types of interventions used: this section explored treatment OT forms such as occupation and activities, intervention to support occupation, education and training, advocacy, and virtual intervention. (4) Perspectives on OBP use including both perceived facilitators (e.g., supportive management, client engagement, and teamwork) and barriers (e.g., limited resources, lack of time, and policy constraints), as well as participants' confidence and perceived effectiveness of OBP.

To ensure content validity, item-objective congruence (IOC) analysis was conducted prior to survey administration. Each item was independently reviewed and scored by three occupational therapy experts with at least 15 years of clinical experience in OBP for clients with physical dysfunctions. Using a scale ranging from −1 to +1 (congruent/agree = +1, questionable = 0, and incongruent/disagree = −1). The questionnaire items with scores greater than or equal to 0.5 were retained. Conversely, items scoring lower than 0.5 with expert comments were then revised according to the suggestions. This revision process was repeated until all items achieved an IOC greater than 0.5. This process ensured that each item aligned with the core domains of OBP practice as conceptualized within the Thai clinical context. To enhance transparency and clarify the questionnaire, a supporting information is included (Appendix), summarizing the key items from each section of the survey and illustrating their alignment with OBP constructs.

The participants were asked to rate their perspective using OBP using a 5-point Likert scale, and the following scale options were used: (1) strongly agree, (2) agree, (3) neutral, (4) disagree, and (5) strongly disagree. The following section presents were the questions asking whether the participants implemented OBP with their clients or if they thought the descriptions were reflective of OBP. The facilitator/barrier portion included the statements about what they thought were facilitators/barriers according to a set of categories or if they were experiencing these facilitators/barriers in their own practice. This questionnaire took approximately 15–20 min to complete.

#### 2.2.4. Interview Participants

The participants were recruited from among occupational therapists who completed the survey and met the following criteria: (1) had more than 3 years of experience using OBP for clients with physical dysfunction and (2) agreed to participate in the interview. We offered them the opportunity to decide whether they were willing to participate in the interviews. After the researchers contacted the participants, they were selected based on the inclusion criteria.

#### 2.2.5. Interview Procedure

The semistructured interview questions were designed based on the results of the quantitative survey (Table 1), which included questions regarding (1) views of OBP, (2) facilitators of using OBP, and (3) barriers to using OBP. The interview process was organized in a logical sequence using a semistructured approach and was conducted via phone.

### 2.3. Data Collection

The survey phase conducted from April 2 to May 3, 2023, involved recruiting participants through electronic posters shared on social media platforms. These platforms included the official Facebook page and website of the Department of Occupational Therapy at Chiang Mai University, as well as the Occupational Therapists Association of Thailand (OTAT) website, which provided a link to a Google Form for responses. Furthermore, the electronic survey was distributed and promoted across hospitals in six regions of Thailand. Since the survey was online, participants with experience using OBP could choose to participate by clicking an electronic informed consent form before starting the survey. At the end of the Google Form survey, participants were asked if they were willing to take part in an individual interview. Those interested could provide their mobile number for the researcher to contact them for the interview phase.

The interview phase was collected from May 15th to July 30th, 2023. The researchers contacted the occupational therapists who indicated that they were interested in being interviewed based on the inclusion criteria. The interview guidelines were designed and refined by an iterative process involving initial mock individual in-depth interviews. Next, the researchers arranged the most convenient time and location for the individual interviews or online interviews via telephone. This was conducted by a researcher following a semistructured interview guideline. Each participant was required to read and sign a written consent form before the interview process, which was audio-recorded and transcribed verbatim. This in-depth interview took approximately 1.5 h to complete. Each step of the research process was clearly documented, and the transcripts were peer-reviewed by the team. Pseudonyms were used to protect the identity of the participants.

### 2.4. Data Analysis

In the survey phase, data was analyzed using frequencies and percentages of descriptive statistics. During this phase, three researchers collaborated to review, analyze, and finalize the results. The interview phase was analyzed by thematic analysis [30]. In this phase, the analysis process involved three researchers and two research assistants. The analytical process consisted of six steps. First, we analyzed the data to obtain a deeper understanding of OTs' experience in OBP. After transcribing the audio verbatim, it was read several times before notes were taken to familiarize ourselves with the data. Second, sections of the text were highlighted, such as phrases or sentences, to describe their content. Throughout the data, codes provided an overview of the main points and groups of common meanings. All interview transcripts were analyzed independently by two coders, both of whom were trained in qualitative research and possessed experience in OBP. Each transcript underwent initial independent coding by two coders to ensure that multiple interpretive perspectives were captured. After that, it dealt with intercoder agreement and consensus procedures. Although a formal intercoder reliability coefficient was not calculated, a consensus-based approach was utilized to ensure coding consistency. Following independent coding, regular meetings were held between the two coders to compare assigned codes, discuss to resolve discrepancies, and refine code definitions. Final codes for each transcript were established through consensus, enhancing analytic rigor and contributing to the credibility of the findings. Third, patterns were identified among the codes and were grouped into potential themes. For example, individual codes such as “limited space,” “use of equipment,” and “areas for doing activities” were merged into the higher-order theme “space and equipment,” which was placed under the main theme “factors influencing the use of OBP.” Fourth, the main themes and subthemes were verified to ensure that they were useful and representative of the data. Fifth, themes were defined and named that shaped the results. Finally, a report was generated based on the research questions and literature.

### 2.5. Rigor and Trustworthiness

To ensure the rigor of the study and adherence to quality standards, the authors employed several strategies to enhance trustworthiness [31]. Purposive sampling, informed by the inclusion criteria, facilitated the selection of participants whose experiences were most relevant to the research context, thereby maximizing the transferability of the findings. Credibility was established through frequent meetings where the authors discussed emerging themes, interpretations, and their understanding of the data [31]. Member checking was performed on the Thai themes and descriptions, including checking again with the English translation. The authors addressed potential translation issues that could impact the reliability of their findings. The detailed transcripts were thoroughly examined on a word-for-word basis in the original Thai language before being translated into English [32]. These translations were subsequently verified by two English-speaking research assistants and a bilingual speaker to ensure accuracy. The rigor and trustworthiness of the emerging themes were supported by the research team analyzing the data independently and agreeing on the themes after analyzing each transcript. Three researchers gathered to discuss and reach a consensus based on a coherent account related to the theme. Furthermore, the research team actively engaged in reflexivity to minimize potential bias throughout the research process [33].

## 3. Results

### 3.1. Survey Outcomes

Seventy-one online questionnaires were returned within the timeframe. However, only 62 participants met the inclusion criteria, had experience with the use of OBP, and fully completed the survey. Those who used OBP worked in both private and public hospitals in Thailand are listed in Table 2. The survey results were analyzed by using frequencies and percentages (descriptive statistics).

**Figure 1:**
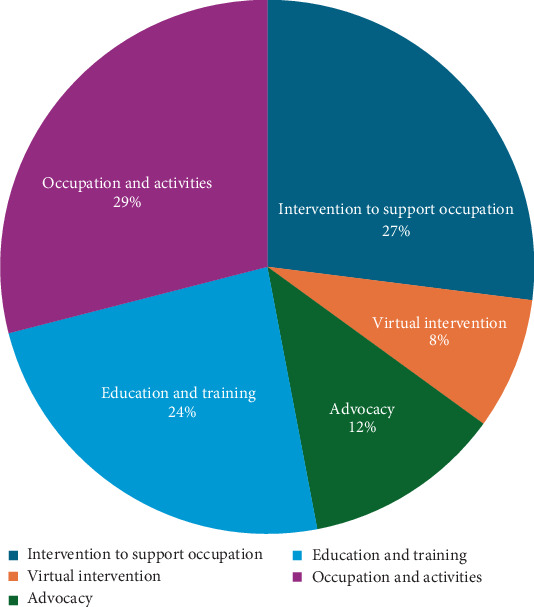
Distribution of the type of occupational therapy intervention among Thai occupational therapists working with physical dysfunction (*n* = 62).

Furthermore, the type of intervention used by the participants is shown as a percentage. The results of this study revealed that 29% of the participants used occupation and activities, 27% used interventions to support their occupation, and 24% used education and training to assist clients with physical dysfunction (Figure 1).

### 3.2. Qualitative Findings

Nine interview participants agreed to participate (three males and six females), and all of them signed consent forms before participating in the study. Pseudonyms were used to protect their identity. The demographic characteristics of the participants are presented in Table 3.

### 3.3. Main Findings (Integrated Quantitative and Qualitative Data)

Two fundamental data issues were presented in the survey: (1) views of OBP and (2) facilitators of and barriers to using OBP. These issues were integrated with two main themes of qualitative findings: (i) attitudes toward occupation driving OBP into action and (ii) factors influencing the use of OBP. Blended numerical and textual data provided the outcomes of the study.

For each key survey outcome, corresponding qualitative insights were presented to interpret the meaning behind the numerical data. These insights include direct participant quotations that illustrate how individual perceptions and experiences reflect broader trends identified in the survey. For instance, survey data indicating widespread agreement that OBP enhances client motivation and engagement is elaborated through qualitative narratives describing increased client participation when therapy is aligned with meaningful, personally relevant tasks. These accounts help to illustrate how such benefits are perceived, internalized, and enacted by therapists in practice.  Table 4 indicates the relationship between each qualitative subtheme and its corresponding quantitative finding. Each subtheme is now explicitly linked to a specific survey result to demonstrate how qualitative data enriches interpretation.

#### 3.3.1. Views of OBP

Participants' views on OBP were organized into three key domains: client-centered goals, OBP implementation, and client motivation and engagement. Overall, the results suggest that OBP is widely embraced in clinical practice. In the domain of client-centered goals, 86% of participants agreed or strongly agreed that clients should be allowed to select occupations that are personally meaningful and contextually relevant. Additionally, 82% felt confident in helping clients achieve their goals through OBP. For OBP implementation, 90% of participants reported providing services based on real-life contexts, and 88% agreed they offered valuable and meaningful occupations as part of their intervention. Furthermore, 87% indicated that they consistently integrated OBP across assessment, treatment, and outcome planning. Regarding motivation and engagement, an overwhelming 95% of therapists agreed or strongly agreed that OBP effectively encourages clients with physical dysfunction to participate more actively in their occupations, as shown in Table 5.

The perspectives of OT participants were supported by qualitative data from the interviews. The purpose was to gain a deeper understanding of the participants' experiences with OBP as a therapeutic approach.

#### 3.3.2. Attitudes Toward Occupation Driving OBP Into Action

The OTs reflected their attitudes toward the use of OBP. In their view, OBP is a fundamental approach in clinical settings, particularly in the field of OT. It revolves around the idea that a person's engagement in meaningful and purposeful occupations, “occupation,” plays a central role in their health and well-being. This theme showed the attitudes of the participants toward OBP in clinical settings from the beginning of the assessment to the intervention for clients. It included two subthemes: perceiving the benefit of OBP for clients and using occupation as the means, as described below.

##### 3.3.2.1. Subtheme: Perceiving the Benefit of OBP for Clients

The main point of this subtheme was the use of OBP for clients because it determines the problems and needs of the client. In addition, OTs perceived this approach as promoting the ability of clients to return to their normal lives. Furthermore, OBP can improve the quality of life of clients. Aran suggested that the benefit of using OBP in clinical settings was related to the occupation of his clients in the following statement.

By using OBP, I am able to better understand the basic daily needs of my clients. It is my responsibility to help them with their basic daily needs. OBPs are based on occupations in the daily lives of clients. This approach can help them return to their real activities and occupations in their contexts. Moreover, it also helps build skills and allows them return to doing what they love. (Aran)

Anong mentioned how OBP encourages clients to continue occupation, which is highlighted in the following interview excerpt:

I saw the benefit of OBP when I trained the hands of my clients for a while. In this case, no additional therapeutic media were available for intervention. Even though the participants improved their hand function after practicing hand activities, they did not continue training since they failed to reach their goals. However, they met their goals while using OBP. By using occupations that suit the needs of the client, I was able to ensure that they would continue their training with me for as long as they wanted by using occupations such as writing or driving, which they actually need for their daily lives. (Anong)

##### 3.3.2.2. Subtheme: Using Occupation as the Means

In clinical settings, it is important to ensure that the occupation chosen for treatment helps clients achieve their goals. The participants needed to assess the occupational performance of the clients before and after treatment related to their goals and needs. The participants must rely on analysis of the activity process, adjust treatment goals, and re-evaluate the clients. In the account of Yada, she planned the treatment goals with her clients relating to their role and context.

I asked the clients about their goals and then planned the treatment with them so that the occupations fit their role and context. The occupation includes their skills required in daily life. I first evaluated the occupational patterns of the clients and then analyzed them. Then, I practiced occupations that matched their abilities to reach their goals. (Yada)

Mukda explained how she planned her goals with her clients and then had them provide feedback on their occupational performance.

I set goals together with my clients. In addition, they provided feedback about their abilities. I always ask my clients to reflect on their occupational performance so that I can analyze and design the occupations that will help them reach their goals. It may or may not be successful all at once. Client satisfaction is determined by his/her performance. (Mukda)

#### 3.3.3. Factors in Using OBP

The factors related to the use of OBP included the client, occupational therapist, policy and service system, and clinical setting and equipment.  Table 6 presents four main factors influencing the use of OBP.

For client-related factors, a large majority of participants agreed (combined agree and strongly agree responses) that OBP should align with clients' health conditions (84%) and roles (79%) and should also align with their motivation for engaging in occupations as well (81%). Regarding the therapist factor, 86% agreed that OBP is an effective treatment method for physical dysfunctions, and 81% supported its use to deliver care within the client's real-life context. However, the agreement was more modest (59%) concerning OBP's impact on improving clients' occupational performance, suggesting variability in perceived outcomes. In the domain of policy and service systems, most participants (60%) disagreed that hospitals lacked policies promoting OBP, and a similar majority (66%) reported insufficient time in the OBP service.

According to the survey findings, most participants viewed client and therapist factors as key facilitators for implementing OBP. In contrast, they identified policy and service system factors, along with clinical settings and equipment, as major barriers to OBP implementation. Hence, these perspectives of OT participants could be supported by qualitative data from the interviews. This purpose revealed deeper insight into the participants' experiences with OBP, focusing on the facilitators of and barriers to its implementation.

#### 3.3.4. Factors Influencing the Use of OBP

Influencing factors that facilitated and/or prevented the participants from using OBP were highlighted here. This theme included three subthemes, namely, partnership among the clients, their relatives, and the interdisciplinary team; organizational management; and space and equipment, as described below.

##### 3.3.4.1. Subtheme: Partnership Among the Clients, Their Relatives, and the Interdisciplinary Team

Communication between clients and therapists is crucial when providing OBP to clients. It is also important for OTs and clients to communicate clearly about their occupational profile, treatment plan, and goals. In addition, OTs can initiate their clients to select and perform relevant activities and meaningful occupations related to their contexts and roles. Furthermore, OTs can design occupations that are adjusted, modified, or applied to meet the needs of their clients. It is important for the interdisciplinary team, the clients, and their relatives to communicate clearly to achieve these goals. Somchai mentioned how he dealt with relatives of the clients through OBP. He makes the following assertion:

During the process, I gradually gained the trust of my clients and their relatives. The activities and occupations they choose for goal setting should be concerned with their values and importance. It can motivate them to engage in these occupations during treatment. (Somchai)

According to Chanthara's statement, “I gradually gained the trust of my clients and their relatives,” which led to building a relationship between clients and relatives and how that influenced a decision on OBP, as highlighted in the following interview extract:

Even though the clients wanted to engage in occupations using OBP, their relatives strongly believed in the medical model, so it was difficult to negotiate with them at the beginning. I understood them because they did not know that meaningful occupations could be effective treatment. It took time for clients to begin performing activities and occupations and accept them as part of their lives. After that, relatives were invited to provide information and help design meaningful activities at home for the clients. (Chanthara)

Sunee proposed a potential solution to address this misunderstanding by embracing teamwork between multidisciplinary teams and the client's family in her private clinic, as stated here:

It is important to work with the client's family as a team in the clinic. I have also worked with doctors, physical therapists, nurses, and nursing assistants. If we do not work together as a team, it is not possible to use the OBP for the clients because they are with me for only 1 hour and because they need time to train with other therapists. If the multidisciplinary team does not understand the OBP, the OBP cannot be successful for those clients. (Sunee)

##### 3.3.4.2. Subtheme: Organizational Management

Providing services to clients requires a multidimensional work management system. Organizational management includes forming a workforce at work, disbursement of the budget for training occupations, and stating the duration of occupational therapy training. The use of OBP can affect decisions made by participants when they apply OBP to individual clients and groups. Preeya mentioned that the workforce is inconsistent with the number of clients. She stated:

In my opinion, the biggest limitations are the time allocations and number of clients. The use of OBP training is based on the characteristics of each client. Therefore, many things must be prepared for practicing occupations with them. Moreover, the number of therapists is not sufficient for training the clients. (Preeya)

Burin raised the issue of inconsistency between the number of clients and the number of therapists in his settings.

The limitation of using OBP was the possibility of more clients receiving OT services than therapists providing them. Therefore, therapists cannot arrange occupations for their clients. For instance, only one therapist was available when eight clients came all at once for OT. It would then be very difficult to train occupations for all eight clients using OBP. I set up group activities at least twice a month or once a week. (Burin)

##### 3.3.4.3. Subtheme: Space and Equipment

By providing OBP, space and equipment can be facilitators or barriers for clients. If there is not enough space or equipment to accomplish certain goals, it may be difficult to achieve this goal with the treatment plan. These factors facilitate or hinder clients from engaging in meaningful and purposeful occupations. Chanthara described that her OT clinical setting was generally a very limited choice for providing meaningful occupations and activities for clients' needs, as the following statement reveals.

There was limited space in hospitals for training clients. It is necessary to share space with other therapists. My OT clinic does not have enough space for clients to perform relevant activities, such as toileting, cooking, grooming, and wheelchair training, because this training requires more space and more equipment. It is a large barrier to allowing clients to do the things they want to do. There are many interventions in a clinic that are very difficult to apply at home or in their environment. (Chanthara)

However, Burin reflected that the use of sharing equipment between clients' workplaces and OT clinical settings can promote the use of OBP for clients, which is highlighted in the following interview excerpt:

There would be no problem with OTs using the OBP if the space is managed well. My client has the opportunity to use equipment from his workplace. My department has a storeroom with enough equipment to support students and clients. (Burin)

## 4. Discussion

In this study, Thai OT participants in various workplaces in Thailand provided their perspectives and experiences using OBP, which were individualized for each client with physical dysfunction. These findings and information provide important contributions to the body of knowledge on the use of OBP. Based on the results, the participants provided occupational therapy services based on OBP derived from real-life contexts of clients (50% strongly agreed and 40% agreed). According to Kaunnil et al. [16], OBP could be applied in Thai contexts (21% strongly agreed and 51% agreed). Their study included occupational therapists from various fields of practice, thereby providing a broad representation of how OBP is integrated into services across various Thai clinical settings. The OTs were aware of how OBP could be used in the Thai hospital system. Furthermore, participants reported providing meaningful occupations through OBP (43% strongly agreed, 45% agreed). Moreover, the use of OBP enables clients with physical dysfunctions to select occupations that align with their individual needs and contexts (47% strongly agreed and 39% agreed). Consistent with Mulligan et al. [34], OTs in New Hampshire were surveyed about determining client goals. Significantly, 93% of respondents reported incorporating occupation-based activities tailored to their clients' needs, emphasizing the high value placed on OBP. In the evaluation process, our study did not explicitly provide questions for participants but implied the implementation of OBP in assessment, treatment, and outcomes for clients with physical dysfunctions (47% strongly agreed and 39% agreed). Hess-April et al. [10] reported that OBP utilizes the assessment phase of intervention planning to inform appropriate interventions. Additionally, Mulligan et al. [34] found that occupation-based assessments ranked among the top five tools used by 53% of respondents, highlighting the significant use of OBP in evaluation methods.

OBP is supported by the beliefs of therapists on the transformational potential of occupations. OBPs are regarded highly by OTs, who emphasize their importance and ensure that they are used regularly and extensively in clinical practice [10]. However, the commitment to OBP and favorable attitudes toward OBP by OTs and their practical implementation of specific therapeutic methods present a conspicuous incongruity. Despite the explicit affiliation of therapists with OBP, this incongruence raises questions about the alignment between professed beliefs and actions [4]. According to this study, occupation, as a philosophical occupational therapy concept and belief, drove OBP into action and showed how to use occupation as a means of reflecting attitudes toward it. This study revealed that the participants viewed OBP as having meaningful and purposeful value as a major benefit.

The qualitative results showed that the participants reflected that OBP can improve their clients' ability to engage in daily life. They utilized OBP to identify their authentic occupations to meet the needs and roles of clients. Moreover, using OBP can assist therapists in guiding clients and their families to identify meaningful occupations that align with their needs and contexts within the treatment program. A systematic review of occupation-based interventions aimed at enhancing occupational performance and participation in hospital settings. This review indicated that OBI could improve occupational performance. However, the evidence was insufficient to conclude whether OBI is more effective than a control or alternative interventions [35]. Consistent with Ngooi et al. [36], a qualitative study on the benefits of occupational therapy activity-based groups highlights their positive impact on patient experience and outcomes, including enhanced independence, motivation, engagement, and therapeutic relationships. In our study, therapists perceived the benefit of OBP for clients with physical dysfunction, using occupation as a means to address individual needs and contexts. To meet these clients' occupation-based goals, it is important to communicate clearly between therapists, clients, and their relatives to promote teamwork in the use of OBP.

In keeping with Estes and Pierce [37], OBPs are more effective and personalized for therapists and more satisfying and rewarding for them. In this study, therapists reported that OBPs were motivating, understandable, valuable, and easily generalized to everyday life for clients and families. According to Hess-April et al. [10], OTs in South Africa found that OBP could inspire and serve as a guiding force to effectively address their clients' needs. They suggested that OTs should emphasize client-centered practices to build good relationships between clients and their families, which can lead to OBP being directed and facilitated by the clients themselves. This study showed that client and therapist factors are related to the use of OBP in clinical settings. A good relationship between the client and therapist can facilitate the effectiveness of OBP treatment.

Robertson [38] highlights the multifaceted nature of clinical reasoning in OT, emphasizing the influence of context on professional thinking. According to Finlay and Gough [39], thinking about one's personal context includes personal knowledge of one's values, knowledge of one's level of professional competence, and awareness of how one's life experiences and life roles may affect how one interprets one's work. Moreover, Ryan and Higgs [40] presented a contextual framework for thinking in clinical reasoning. It guides therapists to consider various contextual factors in their practice. This framework encourages therapists to look at the bigger picture, including social and political policies, community demographics, and service structure. It also emphasizes understanding the team's goals, theoretical approaches, and collaboration with other disciplines. The therapist's background, demographics, and expectations are all crucial aspects for therapists to consider. Ultimately, the framework promotes clinical reasoning to guide individual therapist decisions. Hence, it is believed by the authors that therapists' use of OBP is influenced by several contextual factors of clinical reasoning that orient their reasoning to a specific context of practice, which provides OBP for clients. This comes from participants thinking about individual practices, including their knowledge, beliefs, attitudes, and experiences in using OBP for clients. OBP is an effective way of finding an authentic occupation for clients and providing interventions related to their context. Furthermore, there is a factor that relates to clients and their relatives. In addition, multidisciplinary teams and workplaces can affect decisions made by participants and can be facilitators of or barriers to using OBP in clinical settings.

Certain facilitators and barriers to OBP are inherently tied to the nature of the approach itself, irrespective of geographical or institutional context. The facilitators identified in this study involved mutual understanding between clients and therapists regarding the use of OBP, focusing on clients' abilities, conditions, role performance, and motivation. Therapists need to gain experience in using OBP to enhance the ability of clients with physical dysfunctions to perform their occupations. Consistent with Hess-April et al. [10], this study demonstrated that therapists who are imaginative, adaptable to diverse activities, and well-versed in purposeful activities and meaningful occupations feel more competent in implementing OBP. Likewise, ongoing professional development and access to mentorship programs enhance both skill acquisition and confidence. In this study, therapists reported that initiatives such as in-service training and professional interest groups were essential in maintaining alignment with OBPs [10]. Furthermore, a collegial and supportive work environment is critical. Bolt et al. [19] emphasized the importance of peer collaboration and a culture of shared problem-solving in implementing OBP. Participants likewise indicated that consultation with occupational therapy colleagues, interdisciplinary cooperation, and managerial encouragement collectively promote consistent engagement with OBP. Ultimately, the presence of shared professional values, robust education, and a culture of mentorship intrinsically supports the delivery of occupation-based interventions.

In this study, key barriers to implementing OBP with clients experiencing physical dysfunction included restrictive policies, delayed budget disbursement, limitations in time, space, equipment, and staffing, which were consistently reported across diverse healthcare settings. According to Aas and Bonsaksen [22], lack of time, physical space, and appropriate equipment were all significant impediments to OBP within hospital environments. Similarly, Hess-April et al. [10] emphasized that insufficient funding, material shortages, and excessive caseloads often compel occupational therapists to prioritize expedient, impairment-focused approaches over more holistic OBP. These resource-related constraints such as inadequate personnel, high patient throughput, and the absence of dedicated budgets for occupation-based tools create substantial obstacles to OBP implementation. Therefore, while OBP is intrinsically valuable for promoting client-centered care and professional identity, its effective implementation depends universally on the availability of adequate time, space, equipment, and human resources.

Furthermore, some constraints in the present study appear to be particularly rooted in Thailand's healthcare system. The delivery of OBP is often hindered by institutional structures, insufficient resource allocation, and administrative restrictions. Kaunnil et al. [41] documented how occupational therapists in Thailand encounter excessive bureaucratic procedures, restricted autonomy, and overgeneralized tasks that impede client-centered OBP. Likewise, this study found that 32% of participants strongly agreed and 34% agreed that therapist-to-client ratios significantly affected their capacity to provide OBP. Moreover, tool shortages and restricted session times, attributed to high caseloads and administrative burdens, further constrain implementation. These findings were also in line with Masango et al. [42], who identified policy gaps, ambiguous professional identity, and centralized decision-making as significant impediments to OBP. In Thailand, these challenges are intensified by the urban-centric distribution of therapists, limited interprofessional collaboration, and a broader systemic tendency to undervalue occupational outcomes. Although certain facilitators and barriers are common across contexts, the specific conditions in Thailand introduce additional complexities that demand context-sensitive interventions.

Based on evidence and these results, most OT participants who work with clients with physical dysfunctions believe that OBP effectively enhances clients' abilities with physical dysfunctions by supporting meaningful occupations and facilitating context-based rehabilitation. Key barriers to effective OBP service include insufficient staffing, inadequate facilities and equipment, and unaligned policies that limit OT clinics' ability to achieve occupation-based outcomes for clients. These findings highlight the need for the Ministry of Public Health and the Ministry of Higher Education, Science, Research and Innovation to address therapist staffing ratios, develop an occupation-based curriculum, and enhance facilities and equipment at hospitals and rehabilitation centers.

Despite the Thai government's efforts to decentralize and enhance community health through subdistrict health promoting hospitals and community rehabilitation centers [43], occupational therapist positions remain limited and lack formal integration within organizational structures. With a ratio of only 0.2 OTs per 10,000 population in Thailand [44], substantial policy changes are necessary. The Ministry of Public Health should prioritize the recruitment of OTs for secondary and tertiary hospitals, while the Ministry of Interior should focus on staffing local hospitals and community rehabilitation centers. Moreover, by the year 2038, Thailand is expected to transition into a super-aged society, with older adults comprising 30% of the total population within the next 13 years [45]. It is essential for universities to focus on the education and training of OT students and therapists to effectively meet the needs of people with physical dysfunctions, particularly older adults experiencing physical disabilities and other health challenges that necessitate both short-term and long-term care.

### 4.1. Limitations of the Study

This study faced limitations, including a small sample size restricted by the inclusion criteria, which focused on OTs working specifically with clients with physical dysfunction and possessing experience in the use of OBP. Additionally, the emphasis on OBP as a criterion may have influenced the number of eligible participants, thereby impacting the ability to achieve the intended sample size.

Although the study achieved 62 responses, representing a 72.9% response rate, which is considered acceptable within survey research standards, the final participant count fell short of the initially planned sample size of 85 participants. This discrepancy may have introduced selection bias, as participants who chose to respond may differ systematically in their use of OBP compared to nonrespondents. Nonresponse bias may have influenced findings by inflating OBP usage rates, limiting the diversity of reported barriers and supports, and underrepresenting OTs from certain demographic groups or practice settings. These limitations reduce the representativeness of the sample and restrict the generalizability of the results. Future research should consider employing targeted follow-up methods or mixed recruitment strategies to minimize potential differences between respondents and nonrespondents' experiences and perceptions of OBP. This could reduce nonresponse bias and enhance the representativeness of the data.

The use of convenience sampling and the limited sample size may constrain generalization. This limitation reflected the relatively infrequent adoption of OBP in the treatment of clients with physical dysfunction. The results were consistent with Kaunnil et al. [16] who asserted that most Thai OTs predominantly rely on medical models, with limited integration of frameworks that align with Thai cultural contexts. Consequently, this practice emphasizes body functions and impairments, rather than adopting a holistic, occupation-based approach, impeding the implementation of OBP.

While Thailand is divided into six regions, the therapists who responded to the surveys were not representative of all regions. The study also primarily involved public hospitals; future studies should include more private hospitals and rehabilitation clinics including community rehabilitation centers to achieve a more comprehensive understanding of OBP in clients with physical dysfunction. Future studies should also explore the application of OBP in pediatric and mental health settings.

## 5. Conclusion

This study explored the perspectives and experiences of Thai occupational therapists in implementing OBP with clients who have physical dysfunction. Occupation and meaningful activity were widely recognized as central to therapeutic intervention. Key components of OBP include the development of client-centered goals that reflect individual needs and preferences, the enhancement of client motivation and engagement, and the maintenance of an occupational focus throughout the therapeutic process. A shared emphasis emerged on the importance of adopting a mindset that values participation in everyday occupations as a fundamental therapeutic objective.

Multiple contextual factors were found to influence OBP implementation. These included the characteristics of clients, therapists' own beliefs and competencies, the structure of service delivery and policy frameworks, and the nature of the physical and social clinical environments. Such factors may either support or hinder the integration of OBP into routine practice, highlighting the necessity for systemic and organizational support to facilitate its effective adoption.

Enhancing OBP uptake and sustainability requires strategic changes at both practice and institutional levels. Regular self-reflection and peer mentoring can help embed OBP principles into daily routines, while collaborative goal setting based on meaningful occupations strengthens client engagement. Advocating for the therapeutic value of everyday activities remains essential, particularly in contexts constrained by limited time or resources. At the institutional level, incorporating OBP content into continuing education and entry-level curricula can build foundational competencies. Designing clinical environments that include appropriate space and materials for meaningful activities, along with revising service policies to allow adequate time and flexibility for OBP implementation, can further support long-term integration.

## Figures and Tables

**Table 1:** Examples of interview questions.

**Questions**
1. Can you tell me what OBP is in your opinion? 2. How do you use OBP in your workplace? (client with physical dysfunctions) 3. How do you ensure that the occupations chosen or used in therapy are realistic and consistent with the client's context and role? 4. Can you tell me about the occupations you select or use that have meaningful and purposeful value for your clients? 5. Can you tell me how you ensure your clients' goals will be reached with the occupation you use in therapy? 6. In accordance with OBP approach, how do you support clients in taking, deciding, or choosing an occupation? 7. What has been your experience related to facilitators? How would you describe these facilitators in your practice? 8. What has been your experience related to barriers? How would you describe these barriers in your practice?

**Table 2:** Demographic data of the participants (*n* = 62).

**Participants' practice settings**	**Years of experience**				
	**≥ 1 years**	**2–5 years**	**6–10 years**	**> 10 years**	**Total**
Primary care hospitals	1	1	0	4	6
Secondary care centers/general hospitals	0	6	1	3	10
Tertiary care centers/regional hospitals	2	0	0	0	2
Quaternary care centers/university hospitals	5	3	8	5	21
Private hospitals	5	7	1	0	13
Private clinics	1	1	0	0	2
Others	0	2	2	4	8

**Table 3:** Demographic characteristics of the participants in the individual interviews.

**Participants**	**Gender**	**Regions of Thailand**	**Years of experience in occupational therapy**	**Frequency in using OBP**
Somchai	Male	Northern region	3	Daily
Aran	Male	Central region	7	Daily
Burin	Male	Northeastern region	19	Daily
Sunee	Female	Central region	3	Daily
Mukda	Female	Western region	3	Daily
Anong	Female	Eastern region	5	Daily
Chanthara	Female	Northern region	14	2–3 days/week
Yada	Female	Southern region	16	2–3 days/week
Preeya	Female	Northeastern region	25	Daily

**Table 4:** Integrated findings between quantitative and qualitative outcomes.

**Quantitative method**	**Qualitative method**
*Views of OBP*	*Attitude toward occupation driving OBP into action*
° Client-centered goals (focus on client's needs and desires)	° Perceiving the benefit of OBP for clients
° OBP implementation	° Using occupation as the means
° Motivation and engagement (focus on client's participation)	

*Factors in using OBP*	*Factors influencing the use of OBP*
° Client	° Partnership among the clients, their relatives, and the interdisciplinary team
° Occupational therapist	
° Policy and service system	° Organizational management
° Clinical setting and equipment	° Space and equipment

**Table 5:** Views of OBP (*N* = 62) *n* (%).

**Views of OBP**	**Strongly agree (%)**	**Agree (%)**	**Neutral (%)**	**Disagree (%)**	**Strongly disagree (%)**
*Client-centered goals (focus on client's needs and desires)*					
Allowing clients with physical dysfunctions to choose occupations through OBP that relate to their context	29 (47%)	24 (39%)	6 (9%)	3 (5%)	0 (0%)
Helping clients with physical dysfunction to achieve their goals through OBP	16 (26%)	35 (56%)	10 (16%)	1 (2%)	0 (0%)

*OBP implementation*					
Connecting OBP to assessment, treatment, and outcome for clients with physical dysfunctions	22 (35%)	32 (52%)	6 (10%)	2 (3%)	0 (0%)
Offering valuable and meaningful occupations in OBP for clients with physical dysfunctions	27 (43%)	28 (45%)	6 (10%)	1 (2%)	0 (0%)
Selecting an occupation that meets the occupational performance of clients with physical dysfunctions	20 (32%)	26 (42%)	15 (24%)	1 (2%)	0 (0%)
Providing occupational therapy services based on OBP derived from the real-life contexts of physical dysfunction clients	31 (50%)	25 (40%)	6 (10%)	0 (0%)	0 (0%)
Promoting occupation through OBP to clients with physical dysfunctions	15 (24%)	43 (69%)	4 (7%)	0 (0%)	0 (0%)

*Motivation and engagement (focus on client's participation)*					
Encouraging clients with physical dysfunctions to engage in their occupations through OBP implementation	29 (47%)	30 (48%)	3 (5%)	0 (0%)	0 (0%)

**Table 6:** Factors associated with using OBP (*N* = 62) *n* (%).

**Factors in using OBP**	**Strongly agree (%)**	**Agree (%)**	**Neutral (%)**	**Disagree (%)**	**Strongly disagree (%)**
**1. Client**					
1.1 Utilizing OBP to enable addressing health issues and pathological conditions of clients with physical dysfunctions	23 (37%)	29 (47%)	8 (13%)	2 (3%)	0 (0%)
1.2 Utilizing OBP that aligns with the role of clients with physical dysfunctions	21 (34%)	28 (45%)	11 (18%)	1 (1%)	1 (2%)
1.3 Using OBP with physical dysfunction clients based on their motivation for doing occupation	20 (32%)	30 (49%)	10 (16%)	2 (3%)	0 (0%)
**2. Occupational therapist**					
2.1 Experiencing OBP use to enhance the abilities of physical dysfunction clients in performing occupations	14 (23%)	22 (36%)	22 (35%)	4 (6%)	0 (0%)
2.2 Using OBP as an effective method for treating clients with physical dysfunctions, that is, cerebrovascular disease and spinal cord injuries	22 (36%)	31 (50%)	7 (11%)	2 (3%)	0 (0%)
2.3 Using OBP to allow treatment and rehabilitation in the context of clients with physical dysfunctions	19 (31%)	31 (50%)	11 (18%)	0 (0%)	1 (1%)
**3. Policy and service system**					
3.1 Accessing hospital policies that supported and promoted using OBP for clients with physical dysfunction	1 (1%)	8 (13%)	16 (26%)	24 (39%)	13 (21%)
3.2 Having enough time in the health service system to use OBP for clients with physical dysfunction	2 (3%)	9 (15%)	10 (16%)	21 (34%)	20 (32%)
**4. Clinical setting and equipment**					
4.1 Using clinical settings and spaces to allow application of OBP for clients with physical dysfunction	3 (5%)	4 (6%)	14 (23%)	21 (34%)	14 (23%)
4.2 Having enough occupational therapy equipment and tools for practicing occupations for clients with physical dysfunctions	3 (5%)	7 (11%)	20 (32%)	20 (32%)	12 (20%)

## Data Availability

The data that support the findings of this study are available from the corresponding author upon reasonable request. The data are not publicly available due to privacy or ethical restrictions.

## Supporting Information

Supporting Information Additional supporting information can be found online in the Supporting Information section. Questionnaire on Thai occupational therapists' perspectives and experiences regarding occupation-based clinical practice in the rehabilitation of clients with physical dysfunction. This contains the structured questionnaire used to explore registered Thai occupational therapists' perspectives and experiences using occupation-based clinical practice for clients with physical dysfunction. The instrument includes four parts: (1) respondent demographics and work setting; (2) experience and frequency of using occupation-based practice; (3) types of interventions delivered; and (4) a series of statements rated on a 5-point Likert scale assessing (a) perspectives on OBP (occupation, meaning/goals, therapy purpose, and participation), (b) facilitating factors (client, therapist, organizational, and physical environment), and (c) barriers (client, therapist, organizational, and physical environment).
