# Supplementary material for: Factors Influencing Occupation-Based Practice in Physical Dysfunction: Perspectives of Thai Occupational Therapists
Source: Occup Ther Int. 2025 Dec 3;2025:9955358. doi: 10.1155/oti/9955358 (PMC12695412; doi:10.1155/oti/9955358)
Supplement: Supporting Information — Additional supporting information can be found online in the Supporting Information section. Questionnaire on Thai occupational therapists' perspectives and experiences regarding occupation-based clinical practice in the rehabilitation of clients with physical dysfunction. This contains the structured questionnaire used to explore registered Thai occupational therapists' perspectives and experiences using occupation-based clinical practice for clients with physical dysfunction. The instrument includes four parts: (1) respondent demographics and work setting; (2) experience and frequency of using occupation-based practice; (3) types of interventions delivered; and (4) a series of statements rated on a 5-point Likert scale assessing (a) perspectives on OBP (occupation, meaning/goals, therapy purpose, and participation), (b) facilitating factors (client, therapist, organizational, and physical environment), and (c) barriers (client, therapist, organizational, and physical environment). [file 9955358.f1.docx]

**Appendix A**

**Questionnaire on Thai Occupational Therapists’ Perspectives and Experiences Regarding Occupation-Based Clinical Practice in the Rehabilitation of Clients with Physical Dysfunction**

**Objective:** To explore the perspectives of Thai occupational therapists on clinical practice based on occupation-based concepts in clients with physical dysfunction.

**Participants:** Registered and licensed occupational therapists practicing in the physical dysfunction department.

**Part 1: General Information of the Respondent**

1. **Gender:**
   - Male
   - Female
2. **Age:** _______ years
3. **Education:**
   - Bachelor's Degree
   - Master's Degree
   - Doctorate Degree
   - Other (specify) ___________________________
4. **Workplace:**
   - General Hospital
   - Regional Hospital
   - Private Hospital
   - Community Hospital
   - Other (specify) ___________________________

**Part 2: Experience Using Occupation-Based Practice**

1. **Use of Occupation-Based Practice Concepts**
   - Uses occupation-based practice concepts
   - Does not use occupation-based practice concepts
2. **Experience in Working with Occupation-Based Practice Concepts**
   - Less than 2 years
   - 2-5 years
   - 6-10 years
   - More than 10 years
3. **Frequency of Using Occupation-Based Clinical Practice**
   - Every day
   - 2-3 days/week
   - Once a week
   - 1-2 days/month
   - Other (specify) ___________________________

**Part 3: Types of Intervention**

**Treatment Forms:**

- Occupation and activities
- Intervention to support occupation
- Education and training
- Advocacy
- Virtual intervention

**Part 4: Perspectives on the Use of OBP, Facilitating Factors, and Barriers in Clients with Physical Dysfunction**

**Instructions:** Please consider each statement and mark (/) the box that best reflects your feeling. Please answer all questions using the following 5-level rating scale

- **Strongly Agree:** Means you strongly agree with the statement.
- **Agree:** Means you agree with the statement.
- **Moderately Agree:** Means you moderately agree with the statement.
- **Disagree:** Means you disagree with the statement.
- **Strongly Disagree:** Means you strongly disagree with the statement.

**1. Perspectives on the Use of Occupation-Based Practice (OBP)**

**1.1 Occupation**

| Statement | Strongly Agree | Agree | Moderately Agree | Disagree | Strongly Disagree |
| --- | --- | --- | --- | --- | --- |
| 1.1.1 Occupational therapy services based on occupation-based concepts are derived from the real-life context of clients with physical dysfunction. |  |  |  |  |  |
| 1.1.2 Occupational therapy services based on occupation-based concepts are linked to the assessment, treatment, and outcomes of clients with physical dysfunction. |  |  |  |  |  |

**1.2 Valuing Meaning and Goals**

| Statement | Strongly Agree | Agree | Moderately Agree | Disagree | Strongly Disagree |
| --- | --- | --- | --- | --- | --- |
| 1.2.1 Occupation-based concepts help you find occupations that are valuable and meaningful to clients with physical dysfunction. |  |  |  |  |  |
| 1.2.2 The occupations you use in treatment are consistent with the needs of clients with physical dysfunction. |  |  |  |  |  |

**1.3 Purpose of Occupational Therapy**

| Statement | Strongly Agree | Agree | Moderately Agree | Disagree | Strongly Disagree |
| --- | --- | --- | --- | --- | --- |
| 1.3.1 The occupation-based concepts you choose can help clients with physical dysfunction achieve their set goals for activities. |  |  |  |  |  |
| 1.3.2 Occupation-based concepts help you promote the ability of clients with physical dysfunction to perform daily life activities. |  |  |  |  |  |

**1.4 Participation in Activities**

| Statement | Strongly Agree | Agree | Moderately Agree | Disagree | Strongly Disagree |
| --- | --- | --- | --- | --- | --- |
| 1.4.1 Occupation-based concepts provide opportunities for clients with physical dysfunction to participate in choosing treatment activities that are consistent with their own context. |  |  |  |  |  |
| 1.4.2 Occupation-based concepts help promote the participation of clients with physical dysfunction in daily life activities. |  |  |  |  |  |

**2. Facilitating Factors for the Use of Occupation-Based Practice**

**2.1 Client Factors**

| Statement | Strongly Agree | Agree | Moderately Agree | Disagree | Strongly Disagree |
| --- | --- | --- | --- | --- | --- |
| 2.1.1 You use occupation-based concepts that are consistent with the health condition and pathology of clients with physical dysfunction. |  |  |  |  |  |
| 2.1.2 You use occupation-based concepts that are consistent with the roles of clients with physical dysfunction. |  |  |  |  |  |
| 2.1.3 You use occupation-based concepts that are related to the motivation of clients with physical dysfunction to perform activities. |  |  |  |  |  |

**2.2 Therapist Factors**

| Statement | Strongly Agree | Agree | Moderately Agree | Disagree | Strongly Disagree |
| --- | --- | --- | --- | --- | --- |
| 2.2.1 You have experience using occupation-based concepts to increase the ability of clients with physical dysfunction to perform daily life activities. |  |  |  |  |  |
| 2.2.2 The use of occupation-based concepts is suitable for treatment in clients with physical dysfunction, such as stroke, spinal cord injury, etc. |  |  |  |  |  |
| 2.2.3 Occupation-based concepts help you treat clients with physical dysfunction appropriately according to their context. |  |  |  |  |  |

**2.3 Organizational Factors (Systems Factor)**

| Statement | Strongly Agree | Agree | Moderately Agree | Disagree | Strongly Disagree |
| --- | --- | --- | --- | --- | --- |
| 2.3.1 The organization has policies that support you in using occupation-based concepts with clients with physical dysfunction. |  |  |  |  |  |
| 2.3.2 The organization's health service system facilitates the use of occupation-based concepts with clients with physical dysfunction. |  |  |  |  |  |
| 2.3.3 The health service system provides sufficient time for occupation-based practice with clients with physical dysfunction. |  |  |  |  |  |

**2.4 Physical Environment Factors**

| Statement | Strongly Agree | Agree | Moderately Agree | Disagree | Strongly Disagree |
| --- | --- | --- | --- | --- | --- |
| 2.4.1 The facility and space are conducive to you using occupation-based practice and concepts appropriately for clients with physical dysfunction. |  |  |  |  |  |
| 2.4.2 You have sufficient occupational therapy equipment and tools for training clients with physical dysfunction in daily life activities. |  |  |  |  |  |

**3. Barriers to the Use of Occupation-Based Practice**

**3.1 Client Factors**

| Statement | Strongly Agree | Agree | Moderately Agree | Disagree | Strongly Disagree |
| --- | --- | --- | --- | --- | --- |
| 3.1.1 Clients with physical dysfunction lack understanding of the purpose of treatment using occupation-based concepts. |  |  |  |  |  |
| 3.1.2 Clients with physical dysfunction are uncooperative in treatment using occupation-based concepts. |  |  |  |  |  |
| 3.1.3 Clients with physical dysfunction disagree with the use of occupation-based concepts in occupational therapy treatment. |  |  |  |  |  |

**3.2 Therapist Factors**

| Statement | Strongly Agree | Agree | Moderately Agree | Disagree | Strongly Disagree |
| --- | --- | --- | --- | --- | --- |
| 3.2.1 You have not received training in the use of occupation-based concepts in clients with physical dysfunction. |  |  |  |  |  |
| 3.2.2 You lack opportunities and experience in using occupation-based concepts in clients with physical dysfunction. |  |  |  |  |  |

**3.3 Organizational Factors**

| Statement | Strongly Agree | Agree | Moderately Agree | Disagree | Strongly Disagree |
| --- | --- | --- | --- | --- | --- |
| 3.3.1 The short duration of stay for clients with physical dysfunction in the healthcare facility prevents you from using occupation-based concepts. |  |  |  |  |  |
| 3.3.2 The large number of clients with physical dysfunction in the hospital affects the choice of using occupation-based concepts. |  |  |  |  |  |
| 3.3.3 Other interdisciplinary professionals do not see the value of using occupation-based concepts with clients with physical dysfunction. |  |  |  |  |  |

**3.4 Physical Environment Factors**

| Statement | Strongly Agree | Agree | Moderately Agree | Disagree | Strongly Disagree |
| --- | --- | --- | --- | --- | --- |
| 3.4.1 Insufficient space and facilities for occupation-based clinical practice. |  |  |  |  |  |
| 3.4.2 Insufficient occupational therapy equipment and tools for occupation-based clinical practice. |  |  |  |  |  |
